# Double Transition in Left Bundle Branch Area Pacing

**DOI:** 10.1002/joa3.70290

**Published:** 2026-02-06

**Authors:** Sudipta Mondal, Nadeem Afroz Muslim

**Affiliations:** ^1^ Department of Cardiology The Mission Hospital Durgapur West Bengal India

**Keywords:** double transition, LBBAP, left bundle branch area pacing, selective capture

## Abstract

This case discusses the differential diagnosis of the double transition sign during bipolar threshold testing following conduction system pacing and delves into the details of electrophysiologic parameters of successful left bundle capture.

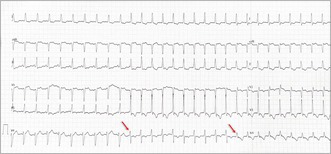

**Abbreviation**
LBBAP: left bundle branch area pacing

A sexagenarian patient underwent Left Bundle Optimized Cardiac Resynchronization Therapy (LOT‐CRT). Left bundle branch area pacing (LBBAP) was established with a 3830 Selectsecure lead (Medtronic, Minneapolis). Cathodal threshold was measured at 0.3 V at 0.5 ms, with the transition from non‐selective to selective capture occurring at an output of 0.5 V.

During bipolar threshold testing, three distinct paced QRS morphologies were noted, indicating sequential changes in capture pattern as the pacing output was reduced (Figure 1). What is the mechanism?

1. First Transition (at 1.5 V; Figure 2A,B): Transition from a “QS” pattern to a “Qr” pattern in lead V1. This transition was attributed to the loss of anodal capture, resulting in a shift from non‐selective bipolar left bundle (LB) capture to non‐selective cathodal LB capture. The corresponding local electrogram (EGM) did not clearly display a distinct left bundle potential due to the simultaneous activation of the left bundle and the local septal myocardium.
2. Second Transition (at 0.5 V; Figure 2B,C): Transition from the “Qr” pattern to a “rSR” pattern in lead V1, followed by complete loss of capture (Figure 2D). This transition was marked by the presence of a deep S wave in the lateral leads. This shift signifies a progression from non‐selective cathodal capture to selective cathodal LB capture, without concurrent local myocardial capture. The Left Ventricular Activation Time (LVAT) was similar (within 10 ms of each other, averaged in multiple beats at paper speed of 200 mm/s) (Figures 3 and 4).
  ○ This pattern led to a resultant right ventricular (RV) activation delay, clinically manifested by a tall R wave in lead V1, a deep S wave in lead I, and the increase in total QRS duration as expected.
  ○ The local EGM at this output demonstrated consistent triple spikes in lead electrogram (Figures 3 and 4). The is probably due to unmasking of LB potential (which is merged with the pacing pulse) due to the lack of local myocardial activation. A careful assessment of ECG showed a very short isoelectric line during selective LB capture as well (Figure 4). However, the precise mechanism underlying this very short (than expected) isoelectric line remains unclear, but it is hypothesized to be due to the early activation of septal myocardial cells by local Purkinje fibers.

**FIGURE 1:**
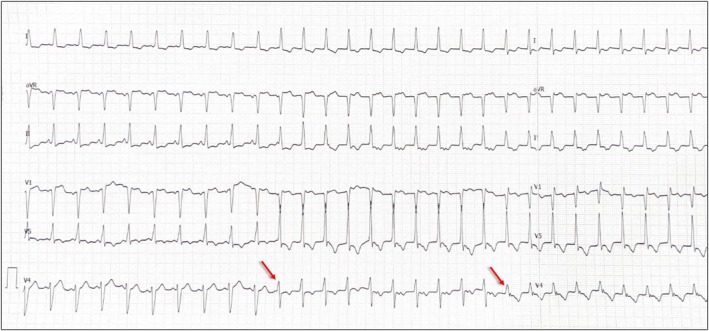
During bipolar threshold testing, three distinct paced QRS morphologies were noted, indicating sequential changes in capture pattern as the pacing output was reduced.

**FIGURE 2:**
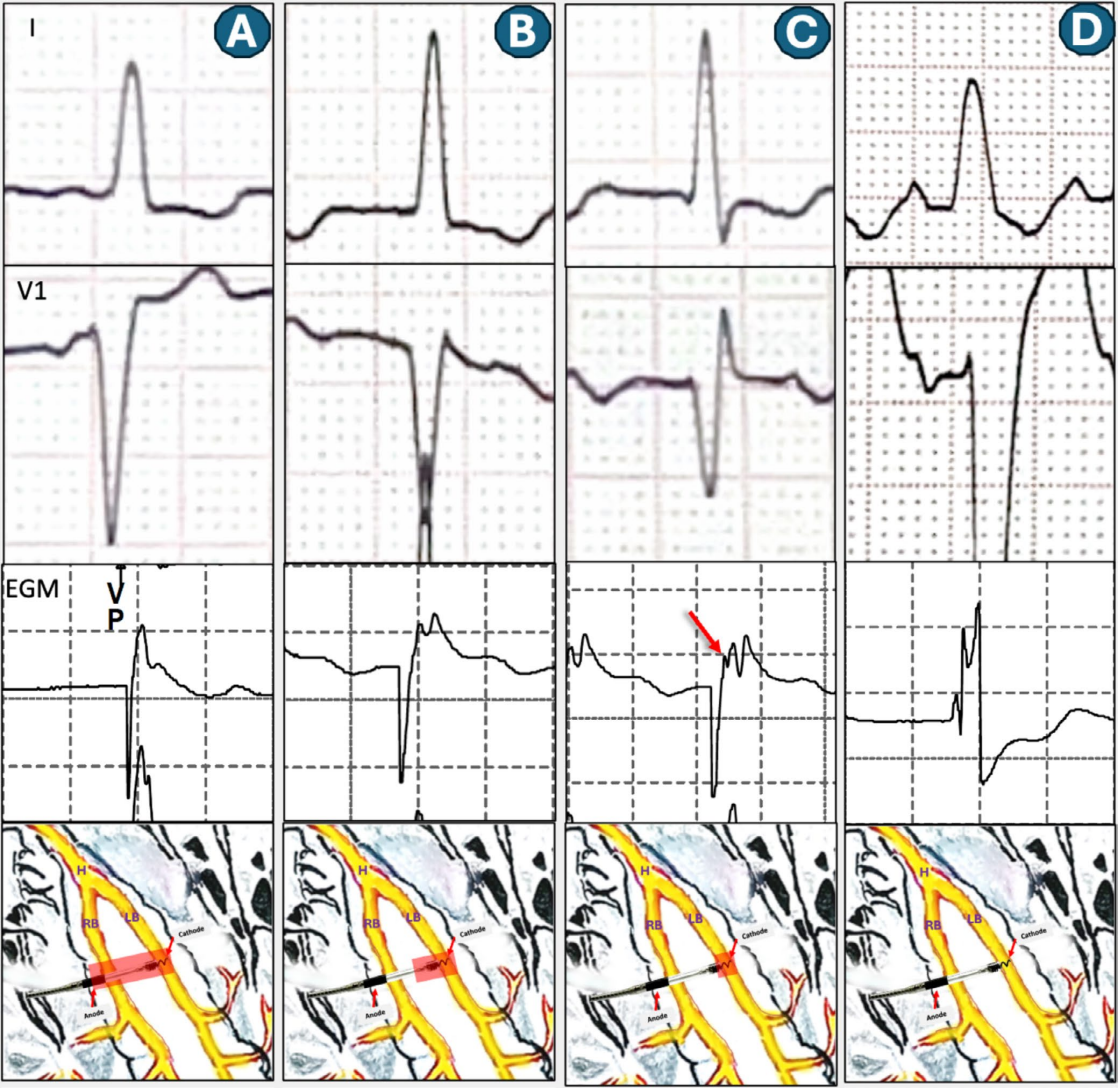
(A, B) Transition (at 1.5 V) from a “QS” pattern to a “Qr” pattern in lead V1. This transition was attributed to the loss of anodal capture, resulting in a shift from non‐selective bipolar left bundle (LB) capture to non‐selective cathodal LB capture. The corresponding local electrogram (EGM) did not clearly display a distinct LB potential due to the simultaneous activation of the left bundle and the local septal myocardium; (B, C) Second Transition (at 0.5 V): Transition from the “Qr” pattern to a “rSR” pattern in lead V1, followed by complete loss of capture (D). This transition was marked by the presence of a deep S wave in the lateral leads and widening of QRS duration (delayed RV activation). This shift signifies a progression from non‐selective cathodal capture to selective cathodal LB capture, without concurrent local myocardial capture (arrow—triple spike, read text for explanation). EGM, electrogram; H, Bundle of His; LB, left bundle; RB, right bundle.

**FIGURE 3:**
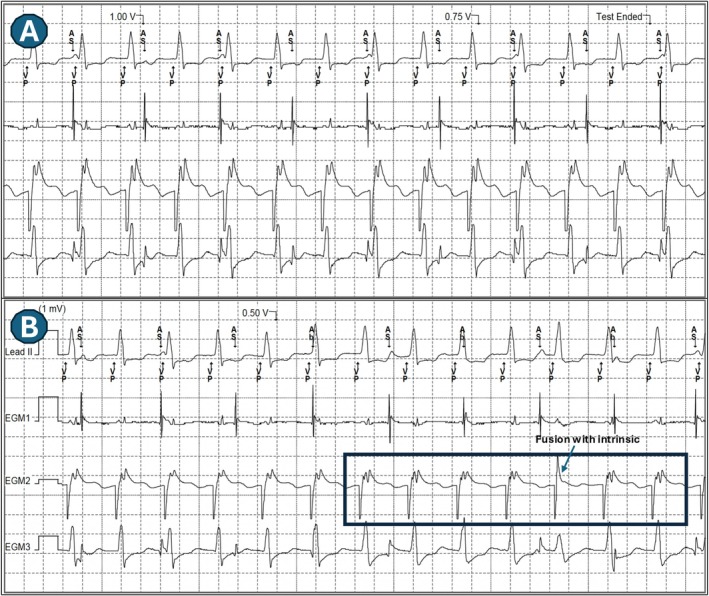
(A) Lead electrogram (EGM) demonstrating non‐selective cathodal capture to selective (B) cathodal left bundle capture. (B) The local EGM at this output demonstrated a consistent triple spikes (box). The is probably due to unmasking of LB potential (which is merged with the pacing pulse) due to the lack of local myocardial activation.

**FIGURE 4:**
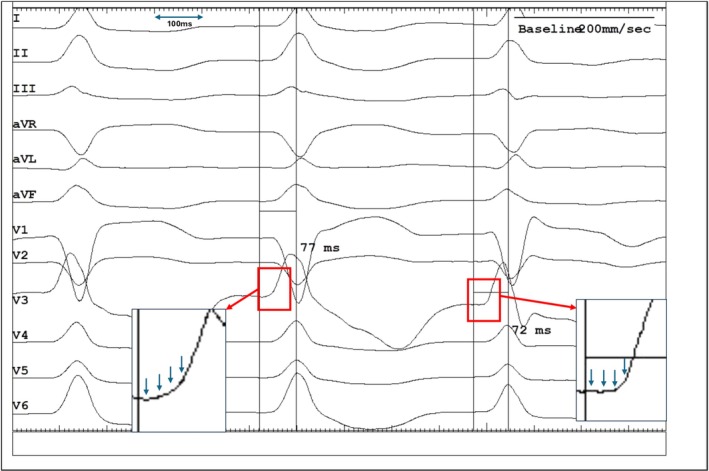
The Left Ventricular Activation Time (LVAT) was similar (within 10 ms of each other, averaged in multiple beats at paper speed of 200 mm/s). A careful assessment of ECG (boxes) showed a very short isoelectric line during selective LB capture as well. However, the precise mechanism underlying this very short (than expected) isoelectric line remains unclear, but it is hypothesized to be due to the activation of septal myocardial cells by local Purkinje fibers. However, given the borderline LVAT and the ambiguity surrounding the isoelectric baseline, LV septal capture must be considered a robust alternative mechanism for the observed transition.

The observed sequence of QRS morphology changes during bipolar threshold testing constitutes a double transition sign. This phenomenon typically reflects two potential capture sequences:

1. Non‐selective bipolar ➔ Non‐selective cathodal LB ➔ Selective cathodal LB capture.
2. Non‐selective bipolar ➔ Non‐selective cathodal LB ➔ Cathodal LV septal capture.

To precisely distinguish between these two possibilities, accurate measurement of the LVAT at the second transition point is critical [1]. An increasing LVAT would suggest a shift to LV septal capture (option 2), while a fixed LVAT would confirm selective cathodal LB capture (option 1).

In the present case, the observed sequence was interpreted as: Non‐selective bipolar ➔ Non‐selective cathodal LB ➔ Selective cathodal LB capture.

The authors agree that the identification of a clear isoelectric line is subjective in this instance. Nevertheless, the emergence of a *probable* isoelectric interval (Figure 4) alongside a *fixed* LVAT supports the hypothesis of Non‐selective ➔ Selective LB capture transition. However, given the borderline LVAT and the ambiguity surrounding the isoelectric baseline, LV septal capture must be considered a robust alternative mechanism for the observed transition.

The presence of the double transition sign during bipolar threshold testing, coupled with a fixed LVAT at the second transition, is highly indicative of an ideal lead position for stable and effective conduction system pacing [2].

## Author Contributions

**Sudipta Mondal and Nadeem Afroz Muslim:** conceptualization (equal), formal analysis (lead), writing – original draft (lead), and writing – review and editing (lead).

## Funding

The authors have nothing to report.

## Ethics Statement

The authors have nothing to report.

## Consent

Obtained from the patient in line with COPE guidance.

## Conflicts of Interest

The authors declare no conflicts of interest.

## Data Availability

All data is incorporated into the article and can be found in online Supporting Information.

## References

[1] H. Wu , L. Jiang , and J. Shen , “Recording an Isoelectric Interval as an Endpoint of Left Bundle Branch Pacing With Continuous Paced Intracardiac Electrogram Monitoring,” Kardiologia Polska 80 (2022): 664–671, 10.33963/KP.a2022.0094. 35380007

[2] S. S. Ponnusamy , “Double Transition Sign‐A Marker of Left Bundle Branch Capture During Physiological Pacing,” Journal of Interventional Cardiac Electrophysiology 65 (2022): 329–330, 10.1007/s10840-021-01109-5. 34985642

